# Supplementary material for: The modulatory effect of Moringa oleifera leaf extract on endogenous antioxidant systems and inflammatory markers in an acetaminophen-induced nephrotoxic mice model
Source: PeerJ. 2016 Jul 7;4:e2127. doi: 10.7717/peerj.2127 (PMC4941779; doi:10.7717/peerj.2127)
Supplement: Data S1 — Raw data compilation [file peerj-04-2127-s001.docx]

|  | **Makmal Hematology & Biokimia Klinikal** | | | |  |  |  |  |
| --- | --- | --- | --- | --- | --- | --- | --- | --- |
|  | KARTHY/Dr. Sharida Fakurazi | | |  |  |  |  |  |
| No. | **Date** | **Lab** | **Sample** | **Creat** | **Urea** | **Na** | **K** | **CL** |
|  |  | **No.** | **ID** | umol/L | mmol/L | mmol/L | mmol/L | mmol/L |
| 1 | 27.6.2013 | R13-117 | PCM | 45 | 15.1 | 149.4 | 12.6 | 106.4 |
| 2 |  | Mice |  | 47 | 17.9 | 143.1 | 11.4 | 107.0 |
| 3 |  |  |  | 43 | 13.4 | 134.2 | 12.9 | 106.9 |
| 4 |  |  | SILYMARIN | 24 | 10.7 | 142.2 | 12.0 | 108.8 |
| 5 |  |  |  | 23 | 10.9 | 139.4 | 11.2 | 105.6 |
| 6 |  |  |  | 26 | 8.9 | 143.4 | 12.0 | 96.6 |
| 7 |  |  | M.O (100) | 26 | 12.4 | 142.6 | 11.0 | 104.0 |
| 8 |  |  |  | 30 | 13.8 | 142.5 | 11.1 | 104.1 |
| 9 |  |  |  | 30 | 14.7 | 140.4 | 12.9 | 105.9 |
| 10 |  |  | M.O (200) | 25 | 9.8 | 140.2 | 10.4 | 103.2 |
| 11 |  |  |  | 23 | 11.4 | 143.7 | 12.0 | 108.3 |
| 12 |  |  |  | 28 | 11.8 | 136.0 | 11.6 | 100.6 |
| 13 |  |  | CONTROL | 22 | 6.5 | 141.0 | 11.2 | 98.8 |
| 14 |  |  |  | 20 | 5.2 | 129.6 | 12.0 | 102.6 |
| 15 |  |  |  | 23 | 9.6 | 143.7 | 10.2 | 107.1 |

Biochemical Parameters:

MDA:

| **GROUPS** | **K** | **K** | **K** |
| --- | --- | --- | --- |
| PCM | 0.638 | 0.648 | 0.663 |
| SILYMARIN | 0.355 | 0.36 | 0.368 |
| M.O (100) | 0.446 | 0.457 | 0.476 |
| M.O (200) | 0.368 | 0.373 | 0.391 |
| CONTROL | 0.327 | 0.364 | 0.352 |

| **Corrected Values / mg of tissue** | | | | |
| --- | --- | --- | --- | --- |
|  | **GROUPS** | **K** |  |  |
|  | PCM | 1.44 |  |  |
|  | SILYMARIN | 0.76 |  |  |
|  | M.O (100) | 0.99 |  |  |
|  | M.O (200) | 0.80 |  |  |
|  | CONTROL | 0.73 |  |  |

SOD:

| **GROUPS** | **K** | **K** | **K** |
| --- | --- | --- | --- |
| PCM | 0.098 | 0.088 | 0.097 |
| SILYMARIN | 0.136 | 0.123 | 0.124 |
| M.O (100) | 0.091 | 0.11 | 0.133 |
| M.O (200) | 0.191 | 0.171 | 0.198 |
| CONTROL | 0.13 | 0.105 | 0.138 |

| **SOD (U/mg)** | | | | |
| --- | --- | --- | --- | --- |
|  |  |  |  |  |
|  |  |  |  |  |
|  |  |  |  |  |
|  | GROUPS | **K** |  |  |
|  | APAP | 47.167 |  |  |
|  | SILYMARIN | 63.833 |  |  |
|  | M.O (100) | 55.667 |  |  |
|  | M.O (200) | 93.333 |  |  |
|  | CONTROL | 62.167 |  |  |

CAT:

| **GROUPS** | **K** | **K** | **K** |
| --- | --- | --- | --- |
| **PCM** | 2.285 | 2.24 | 2.136 |
| **SILYMARIN** | 3.389 | 3.575 | 3.251 |
| **M.O (100)** | 2.515 | 2.937 | 3.009 |
| **M.O (200)** | 2.647 | 2.573 | 3.891 |
| **CONTROL** | 3.462 | 3.408 | 3.358 |

| **CAT activity** | | | | |
| --- | --- | --- | --- | --- |
|  |  |  |  |  |
|  |  |  |  |  |
|  |  |  |  |  |
|  | **GROUPS** | **K** |  |  |
|  | **PCM** | 65.834 |  |  |
|  | **SILYMARIN** | 104.268 |  |  |
|  | **M.O (100)** | 85.300 |  |  |
|  | **M.O (200)** | 92.329 |  |  |
|  | **CONTROL** | 104.408 |  |  |

GPX

|  |  |  |  |  |  |
| --- | --- | --- | --- | --- | --- |
| **GROUPS** | **K** | **K** | **K** |  |  |
| **PCM** | 0.384 | 0.361 | 0.397 |  |  |
| **SILYMARIN** | 0.519 | 0.446 | 0.475 |  | 1 MIN |
| **M.O (100)** | 0.479 | 0.45 | 0.469 |  |  |
| **M.O (200)** | 0.561 | 0.605 | 0.598 |  |  |
| **CONTROL** | 0.369 | 0.495 | 0.439 |  |  |
|  |  |  |  |  |  |
|  |  |  |  |  |  |
| **GROUPS** | **K** | **K** | **K** |  |  |
| **PCM** | 0.384 | 0.358 | 0.396 |  | 2 MIN |
| **SILYMARIN** | 0.516 | 0.445 | 0.375 |  |  |
| **M.O (100)** | 0.476 | 0.448 | 0.468 |  |  |
| **M.O (200)** | 0.56 | 0.601 | 0.595 |  |  |
| **CONTROL** | 0.537 | 0.492 | 0.513 |  |  |
|  |  |  |  |  |  |
|  |  |  |  |  |  |
| **GROUPS** | **K** | **K** | **K** |  |  |
| **PCM** | 0.381 | 0.357 | 0.394 |  |  |
| **SILYMARIN** | 0.513 | 0.443 | 0.373 |  | 3 MIN |
| **M.O (100)** | 0.474 | 0.447 | 0.466 |  |  |
| **M.O (200)** | 0.56 | 0.596 | 0.594 |  |  |
| **CONTROL** | 0.537 | 0.491 | 0.512 |  |  |
|  |  |  |  |  |  |
|  |  |  |  |  |  |
|  |  |  |  |  |  |
| **GROUPS** | **K** | **K** | **K** |  |  |
| **PCM** | 0.383 | 0.358 | 0.394 |  |  |
| **SILYMARIN** | 0.512 | 0.444 | 0.372 |  | 4 MIN |
| **M.O (100)** | 0.473 | 0.448 | 0.463 |  |  |
| **M.O (200)** | 0.557 | 0.598 | 0.592 |  |  |
| **CONTROL** | 0.538 | 0.489 | 0.51 |  |  |

| **GROUPS** | **K** |
| --- | --- |
| PCM | 2.547 |
| SILYMARIN | 6.792 |
| M.O (100) | 6.792 |
| M.O (200) | 7.641 |
| CONTROL | 4.245 |

TNF – alpha

| **GROUPS** | **Kid** | | | **Std** | |
| --- | --- | --- | --- | --- | --- |
| **PCM** | 0.467 | 0.363 | 0.35 | 1.367 | 1.272 |
| **SILYMARIN** | 0.348 | 0.296 | 0.279 | 0.727 | 0.982 |
| **M.O (100)** | 0.402 | 0.324 | 0.309 | 0.465 | 0.473 |
| **M.O (200)** | 0.349 | 0.276 | 0.257 | 0.293 | 0.288 |
| **CONTROL** | 0.124 | 0.157 | 0.218 | 0.147 | 0.157 |
|  |  |  |  | 0.088 | 0.098 |
|  |  |  |  | 0.07 | 0.072 |
|  |  |  |  |  |  |
| **GROUPS** | **Kid** | | |  |  |
| **PCM** | 508.75 | 378.75 | 362.50 | **416.67** |  |
| **SILYMARIN** | 360.00 | 295.00 | 273.75 | **309.58** |  |
| **M.O (100)** | 427.50 | 330.00 | 311.25 | **356.25** |  |
| **M.O (200)** | 361.25 | 270.00 | 246.25 | **292.50** |  |
| **CONTROL** | 80.00 | 121.25 | 197.50 | **132.92** |  |

IL-1Beta

| **GROUPS** | **Kid** | | | **Std** | |
| --- | --- | --- | --- | --- | --- |
| **PCM** | 0.403 | 0.369 | 0.383 | 1.31 | 1.242 |
| **SILYMARIN** | 0.166 | 0.248 | 0.205 | 0.688 | 0.739 |
| **M.O (100)** | 0.291 | 0.281 | 0.334 | 0.454 | 0.457 |
| **M.O (200)** | 0.166 | 0.178 | 0.216 | 0.265 | 0.289 |
| **CONTROL** | 0.103 | 0.123 | 0.125 | 0.094 | 0.092 |
|  |  |  |  | 0.086 | 0.092 |
|  |  |  |  | 0.094 | 0.095 |
|  |  |  |  |  |  |
|  |  |  |  |  |  |
| **GROUPS** | **Kid** | | |  |  |
| **PCM** | 266.50 | 238.17 | 249.83 | **251.50** |  |
| **SILYMARIN** | 69.00 | 137.33 | 101.50 | **102.61** |  |
| **M.O (100)** | 173.17 | 164.83 | 209.00 | **182.33** |  |
| **M.O (200)** | 69.00 | 79.00 | 110.67 | **86.22** |  |
| **CONTROL** | 16.50 | 33.17 | 34.83 | **28.17** |  |

IL-6

| **GROUPS** | **Kid** | | | **Std** | |
| --- | --- | --- | --- | --- | --- |
| **PCM** | 0.575 | 0.541 | 0.538 | 1.219 | 1.058 |
| **SILYMARIN** | 0.428 | 0.391 | 0.389 | 0.468 | 0.404 |
| **M.O (100)** | 0.511 | 0.5 | 0.497 | 0.359 | 0.313 |
| **M.O (200)** | 0.478 | 0.379 | 0.376 | 0.232 | 0.166 |
| **CONTROL** | 0.345 | 0.285 | 0.292 | 0.128 | 0.124 |
|  |  |  |  | 0.098 | 0.109 |
|  |  |  |  | 0.092 | 0.073 |
|  |  |  |  |  |  |
| **GROUPS** | **Kid** | | |  |  |
| **PCM** | 463.27 | 432.36 | 429.64 | **441.76** |  |
| **SILYMARIN** | 329.64 | 296.00 | 294.18 | **306.61** |  |
| **M.O (100)** | 405.09 | 395.09 | 392.36 | **397.52** |  |
| **M.O (200)** | 375.09 | 285.09 | 282.36 | **314.18** |  |
| **CONTROL** | 254.18 | 199.64 | 206.00 | **219.94** |  |

IL-10

| **GROUPS** | **Kid** | | | **Std** | |
| --- | --- | --- | --- | --- | --- |
| **PCM** | 0.421 | 0.417 | 0.427 | 2.689 | 1.659 |
| **SILYMARIN** | 0.456 | 0.449 | 0.465 | 2.137 | 2.192 |
| **M.O (100)** | 0.463 | 0.459 | 0.479 | 1.294 | 1.259 |
| **M.O (200)** | 0.604 | 0.598 | 0.596 | 0.606 | 0.661 |
| **CONTROL** | 0.497 | 0.473 | 0.495 | 0.355 | 0.49 |
|  |  |  |  | 0.221 | 0.267 |
|  |  |  |  | 0.095 | 0.093 |
|  |  |  |  |  |  |
| **GROUPS** | **Kid** | | |  |  |
| **PCM** | 130.71 | 128.81 | 133.57 | **131.03** |  |
| **SILYMARIN** | 147.38 | 144.05 | 151.67 | **147.70** |  |
| **M.O (100)** | 150.71 | 148.81 | 158.33 | **152.62** |  |
| **M.O (200)** | 217.86 | 215.00 | 214.05 | **215.63** |  |
| **CONTROL** | 166.90 | 155.48 | 165.95 | **162.78** |  |
